# Understanding reasons for delay in diagnosis of leprosy in Pakistan: A qualitative study

**DOI:** 10.1371/journal.pntd.0012764

**Published:** 2025-01-07

**Authors:** Anil Fastenau, Maxwell Oliver Beresford, Matthew Willis, Sophie CW. Stuetzle, Fabian Schlumberger, Heleen Neeltje Willemijn Duighuisen

**Affiliations:** 1 Marie Adelaide Leprosy Center, Karachi, Pakistan; 2 German Leprosy and Tuberculosis Relief Association (DAHW), Wuerzburg, Germany; 3 Heidelberg Institute of Global Health, University of Heidelberg, Heidelberg, Germany; 4 Department of Global Health, Institute of Public Health and Nursing Research, University of Bremen, Bremen, Germany; 5 School of Medicine, Dentistry & Biomedical Sciences, Queen’s University Belfast, Belfast, United Kingdom; Yale University School of Medicine, UNITED STATES OF AMERICA

## Abstract

**Background:**

Recent epidemiological data shows significant rates of grade 2 disability at point-of-diagnosis among new leprosy cases in Pakistan. This indicates a feature of extensive diagnostic delay; the disability burden appears unmoving and disproportionate to the falling leprosy incidence rates. Therefore, this study was required to understand reasons for delay in diagnosis and treatment of leprosy.

**Methods:**

A qualitative design of 7 semi-structured interviews was employed to reveal perceptions and understandings of various leprosy stakeholders in Pakistan, termed “leprosy experts”. Subsequent inductive analysis was used to identify themes and subthemes concerned with delay in the diagnosis and treatment of leprosy.

**Results:**

Leprosy experts identified three main areas, or domains, to which delay can be attributed: 1. Awareness and beliefs about leprosy, within the general population, 2. Knowledge and clinical experience of leprosy, among healthcare professionals, 3. Leprosy control program infrastructure, allocation of resources and institutional funding. These domains were each viewed as consequent to the larger theme of ‘low-endemicity’. Strong correlations between diagnostic delay and socioeconomic status, gender, geography and health system challenges, were also mentioned, and which intersected the three major themes.

**Conclusion:**

Reasons for diagnostic delay are evident in all tiers of the healthcare hierarchy in Pakistan. Thus, an approach at multiple levels is justified, to improve the general awareness of leprosy, education of healthcare professionals, and organizational structuring. Additionally, cultural features relevant to different communities in Pakistan which might be different from other care access frameworks demonstrated a need for further study into the health beliefs of Pakistani patients in a wide range of communities.

## Author summary

This study addresses the persistent issue of diagnostic delay in leprosy cases in Pakistan, where grade 2 disability remains prevalent at the point of diagnosis, despite declining incidence rates. Through qualitative analysis, seven semi-structured interviews with key stakeholders, termed "leprosy experts," were conducted to explore the underlying factors contributing to these delays.

The findings identified three primary domains responsible for the diagnostic delay: (1) low awareness and misconceptions about leprosy in the general population, (2) inadequate knowledge and clinical experience of leprosy among healthcare professionals, and (3) deficiencies in the leprosy control program, including infrastructure, resources, and funding. These factors were found to be influenced by the broader context of low-endemicity.’ Additionally, correlations between diagnostic delays and variables such as socioeconomic status, gender, geography, and systemic health challenges were highlighted.

The study concludes that diagnostic delays in leprosy affect all levels of the healthcare system in Pakistan. Therefore, a multi-faceted approach is necessary, involving raising public awareness, enhancing healthcare professionals’ education, and improving organizational structures. The cultural differences within Pakistan also warrant further research to better understand the health beliefs and access barriers in diverse communities.

## Introduction

Leprosy, also known as Hansen’s disease, is a neglected tropical disease caused by Mycobacterium leprae that predominantly affects the skin and peripheral nervous system [1]. If not diagnosed and treated promptly, leprosy can lead to severe irreversible disabilities [2]. However, through accurate early case identification and a strict multidrug therapy (MDT) treatment, leprosy can be easily treated without any physical consequences [3]. Even though, for the past few decades global leprosy reduction strategies have presented the path towards elimination as straightforward, leprosy remains one of the leading causes of preventable disabilities globally [4]. More than 3 million people are affected by leprosy-related disabilities worldwide [5]. Thus, delay of diagnosis among new leprosy cases remains one of the major challenges for many countries in achieving WHO target of Zero Leprosy 2030 [6]. Therefore, early detection of new leprosy cases is crucial for preventing disabilities and for interrupting transmission to help achieve leprosy elimination [7].

Pakistan is classified as a low-endemic country with approximately 250–275 new leprosy cases annually [8]. Despite significant drops in national leprosy burden, the high incidence of new leprosy cases with grade 2 deformities (G2D) indicates the current challenges of delay of diagnosis in Pakistan [9]. G2D frequently serves as a sign of delayed diagnosis, with a high prevalence of cases exhibiting visible deformities suggesting substantial diagnostic delays within a population. Delay of diagnosis in individuals affected by leprosy is defined “delay was defined as the time between the onset of first symptoms related to leprosy, and a correct medical diagnosis”. Extended diagnostic delays often result in peripheral nerve damage, ultimately leading to visible deformities classified as grade 2 disability (G2D) according to the WHO classification system.

The factors contributing to delays in leprosy case detection globally are multifaceted and context-dependent, including sociodemographic characteristics, health-seeking behavior, and the performance of the health system [5,10–12]. Addressing these delays requires a comprehensive understanding of these factors to inform effective public health strategies. However, to the best of our knowledge no studies have researched the delay in leprosy diagnosis within Pakistan. Until now, no in-depth qualitative research has been conducted exploring the reasons behind the delay in leprosy diagnosis in the country. Identifying specific barriers to accessing appropriate leprosy care remains under-investigated, and more public health challenges likely lie before this diagnostic barrier. Therefore, this study aims to address this research gap by exploring the perceptions and experiences of leprosy health professionals with the delay of leprosy diagnosis in Pakistan. A deeper understanding of the reasons and underlying factors of diagnostic delay may assist in developing a strategy to improve early diagnosis and interruption of transmission, which could consequently prevent disability among newly detected leprosy cases and support potential road maps towards zero leprosy in Pakistan [13].

## Methods

### Study design

The study employed a qualitative method to explore the perceptions and experiences of leprosy experts with diagnostic delay in leprosy within the Pakistani healthcare system. The methodology of semi-structured leprosy expert interviews was chosen as it provided flexibility, allowing for in-depth perspectives on leprosy diagnosis in Pakistan, uncovering broader cultural insights related to health, stigma, and societal perceptions beyond just delayed diagnosis and leprosy. The study was conducted online due to geographical constraints, with interviews arranged with participants associated with the Marie Adelaide Leprosy Centre (MALC) and Aid to Leprosy Patients (ALP). Interviews were conducted via the online conference platform Zoom in English and recorded following participants consent. This approach allowed for a diverse range of insights from different geographical locations within Pakistan, which would have been logistically challenging with face-to-face interviews.

### Study setting

This study was focused on the whole country of Pakistan. Pakistan consists of four provinces: Punjab, Khyber Pakhtunkhwa, Sindh, and Balochistan, along with three territories: Islamabad Capital Territory, Gilgit-Baltistan, and Azad Kashmir [14]. Pakistan has no official national leprosy control program. The leprosy control activities are mainly implemented by two Pakistani civil society NGOs: ALP and MALC. ALP is responsible for the leprosy control activities in the province of Punjab, including the Islamabad Capital Territory and territories of Azad Kashmir. While MALC covers the leprosy control activities in the rest of Pakistan.

### Study population

The study population consisted of leprosy health professionals (leprosy experts) from the two main Pakistani NGOs, ALP and MALC which run the de facto national leprosy program, who have long-term experience in working with leprosy within the Pakistani context. ‘Leprosy experts’ as the categorization used in inclusion criteria refers to various kinds of healthcare workers, dermatologists and other doctor specialties, as well as field officers, managers and directors for various leprosy control projects. 85 percent of the experts have more than twenty years of field experience in diagnosing and treating leprosy and are therefore frontline health workers. Demographic details of the interviewees are summarised below in Table 1.

**Table 1:** Participant demographics.

Variables		Number of participants n = 7
Sex	Male	5
	Female	2
Profession	Medical director	1
	Leprosy Field officer	2
	Medical doctor	2
	Statistician	1
	Paramedic	1
Years of experience with leprosy in Pakistan	7–10	2
	10–20	0
	20–30	1
	30–40	4
Organization	ALP	2
	MALC	5

### Data collection

Semi-structured interviews were used, guided by a questionnaire that directed the conversation towards themes around interviewee background, access to healthcare, and diagnostic trajectories (S1 File). This interview guide was prepared in advance and based on Levesque’s framework of accessing healthcare and already existing interview guides of similar research on diagnostic delay of leprosy [16]. It was evaluated by experts in the field of leprosy research. 7 leprosy experts were contacted by email and all 7 replied, the experts were chosen due to having a predetermined minimum of 5 years leprosy work experience in Pakistan–with the actual lowest number of years’ experience included in the study being 7 years. The in-depth interviews aimed to reveal expert perceptions on patient health access and reasons for not seeking care, as well as aspects of the healthcare system that contribute to delays.

### Data analysis

Interviews were transcribed and thematically analysed using Braun and Clarke’s method [15]. The thematic analysis approach is well-suited for identifying patterns within qualitative data, providing a structured yet flexible way to interpret complex information. Data was coded using Atlas.ti software, identifying and organizing key themes, concepts, and ideas. Initially, data were grouped into eight broad categories: personal beliefs and awareness among the public, professional knowledge and expertise, health infrastructure organization, socioeconomic status, gender, culture, health system challenges and geographical barriers. After thematic analysis was completed, Levesque’s framework was used to structure and display the different reasons for delay, which is reflected in the results section [16].

### Triangulation

The participants were closely involved in active case finding and diagnosis, representing a range of medical professions. Thematic saturation was achieved through data triangulation. One researcher conducted the interviews, while three researchers developed the interview guide and two verified the data analysis. The results, structured using Levesque’s framework, were discussed by the entire research team.

### Ethics statement

Ethical clearance was sought from the Ethics Committee of Marie Adelaide Leprosy Centre (MAC/ERC/2023/3). Informed consent was obtained from all participants using the form in (S2 File), and anonymity was maintained during transcription. Data was stored securely and confidentially, with identifying information removed from transcripts.

## Results

**Fig 1:**
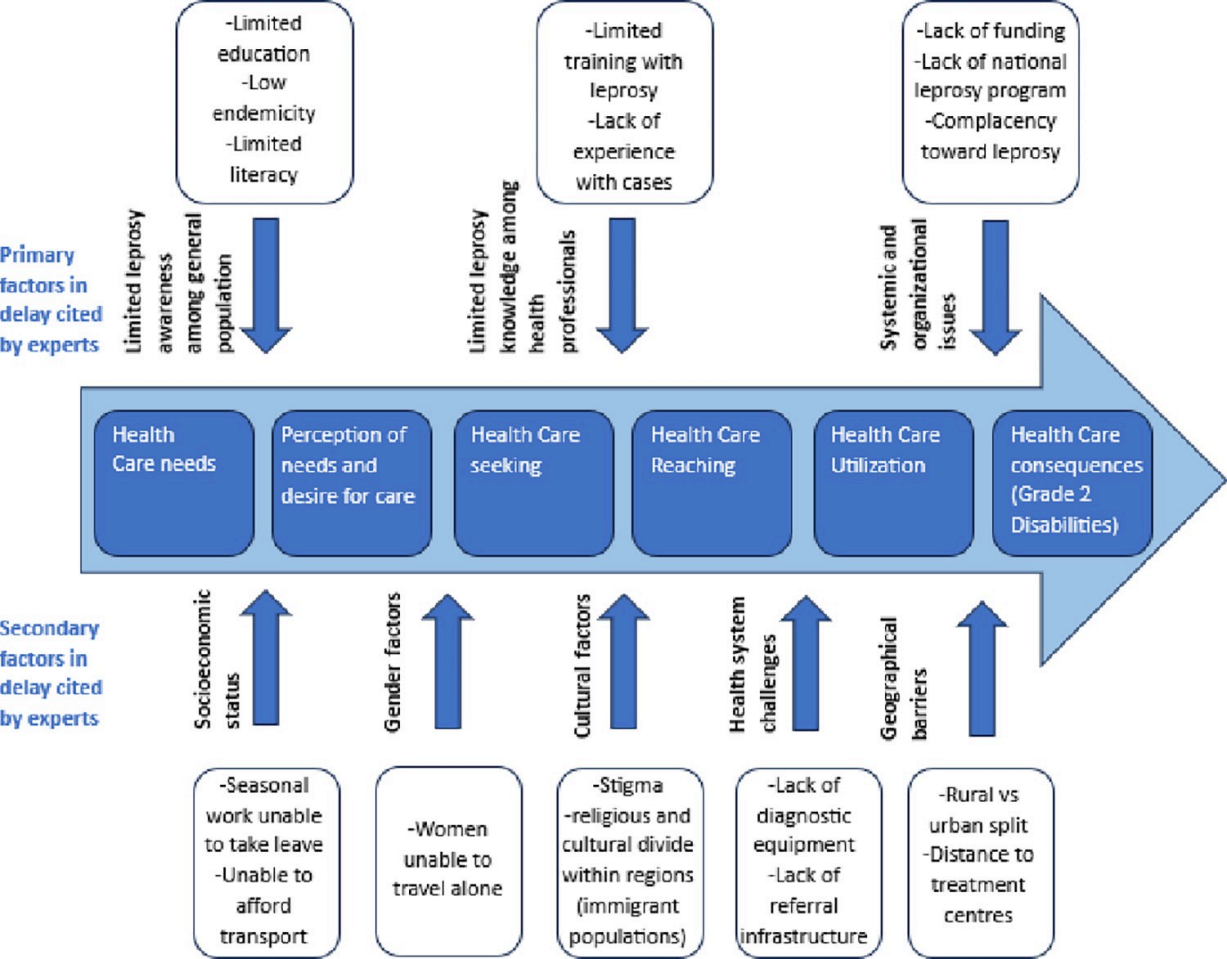
Reasons for diagnostic delay of leprosy in Pakistan.

Based on the interview results, eight reasons for diagnostic delay of leprosy in Pakistan were identified, separated into 3 primary reasons and 5 secondary reasons for delay: (1) Limited awareness among the population, (2) limited knowledge and expertise of healthcare professionals, (3) and issues in leprosy control program infrastructure related to resource allocation and institutional funding were considered the three main reasons for delay according to the leprosy experts. Socioeconomic status, gender, culture, health system challenges and geographical barriers were also mentioned, however, not being explicitly stated as primary causes of delay. Below, the various reasons for diagnostic delay are organized and displayed in an adapted model of Levesque et al’s framework (Fig 1) [16,17]. The model shows the five dimensions of healthcare access and the consequence–in this case delayed diagnosis leading to higher incidence of grade 2 disabilities. The components within the arrow demonstrate the route leading to healthcare access and the elements outside the arrow illustrate the various reasons for delay within each category.

### Awareness and beliefs about leprosy within the general population

The interviews identified that the general population’s awareness and beliefs about leprosy significantly contribute to diagnostic delays. Misconceptions about leprosy, stigma, and lack of knowledge about symptoms often prevent individuals from seeking timely medical attention–cited to be particularly prevalent in migrant communities, making passive case finding difficult. Educational campaigns and public health interventions are needed to dispel myths and encourage early medical consultation.

*“In general, there’s not very good public knowledge about leprosy, so people are not that aware. And so, I’ve been surprised at how much people just tend to ignore the symptoms of leprosy and come very late.” (3, Medical doctor)*

Although many experts identified knowledge of leprosy as being poor among the general population, possibly due to low prevalence, stigma does exist among groups with higher knowledge and higher local prevalence of leprosy. A common belief reported by our expert interviews is that some communities still view leprosy as a highly contagious and untreatable disease, which fosters fear and social stigma. Such beliefs are deeply rooted in cultural and religious contexts, where leprosy might be seen as divine retribution for moral failings. This stigma leads to social exclusion from societal groups, with affected individuals hiding their symptoms or avoiding seeking medical help to prevent ostracism from their communities. This delay can result in severe complications and disabilities by the time medical intervention is sought. Furthermore, this is a major challenge in the interruption of transmission and elimination of leprosy within specific communities with established stigma. One such group identified was Indian migrants from high endemic areas in India where leprosy and therefore leprosy stigma are more prevalent. This was pointed out by the following participant:

*“Only Indians and the people who are more educated […] or the people with very high-profile families, they have stigma. But the other people they don’t have a stigma. […] People who know leprosy, they think that it is very infectious, and if we make some relation with them, we will get leprosy.” (7, Paramedical worker)*

Multiple interviews raised the importance of public health education as crucial in addressing these issues. Effective educational campaigns need to target various segments of the population, using culturally appropriate messages to challenge myths and provide accurate information about the disease, its transmission, and treatment. Community engagement through local religious leaders and political influencers was also suggested to change public perceptions and encourage early diagnosis and treatment.

### Knowledge and clinical experience of leprosy among healthcare professionals

Healthcare professionals’ knowledge and clinical experience with leprosy are critical in early diagnosis and treatment. However, our study found that interviewees believed many healthcare workers in Pakistan lack adequate training and experience with leprosy, leading to misdiagnosis or delayed diagnosis. Continuous medical education and training programs related to leprosy are essential to equip healthcare providers with the necessary skills and knowledge to identify and manage leprosy cases effectively.

Healthcare providers are often the first point of contact for patients with leprosy symptoms. Therefore, their ability to recognize the signs and symptoms of leprosy is crucial. The study revealed perceived gaps in the training and clinical experience of healthcare workers, many of whom had limited exposure to leprosy cases. This lack of familiarity can lead to misdiagnosis, where leprosy symptoms are mistaken for other skin conditions, delaying appropriate treatment. One participant illustrated this as follows:

*“Primary health care staff has no understanding about skin diseases, it is that all of the primary health care staff basically is guessing, they simply prescribe something for the satisfaction of a patient without making a proper diagnosis, or without understanding what the issue could be.” (1, Medical director)*

To address this, the experts felt continuous professional development programs are needed to enhance the diagnostic skills of healthcare providers. They felt such programs should include detailed training on the clinical presentation of leprosy, differential diagnosis, and the use of diagnostic tools. Incorporating leprosy education into the medical curriculum and providing regular “refresher” courses can ensure that healthcare providers remain updated on the latest practices in leprosy management.

*“The most critical part is being played by the doctor who first sees a patient, because they also should be well aware that when they suspect a patient with leprosy, first thing is to suspect, and then to properly refer them to the proper facility.” (4, Medical doctor)*

### Leprosy control program infrastructure, resource allocation and institutional funding

The national program infrastructure and allocation of resources was cited as playing a crucial role in diagnostic delays. The study identified several systemic issues such as insufficient funding for leprosy programmes, lack of specialized facilities, and inadequate coordination between different levels of the healthcare system. The following participate elaborates on this:

*“There is no national leprosy programme. I mean, the government is not doing anything about it, although it is a responsibility of the government. So, it is an organisational programme, there are these two NGOs. […] it is not a core health issue in Pakistan, so they are not taking it as a priority.” (7, Paramedical worker)*

Multiple interviewees also raised concerns around the complacency of the disease status of leprosy in Pakistan, as pointed out by the following participant:

*“Main barrier is that people might be thinking, both the health force and the community, that leprosy has been eradicated, there are very few patients who might have this issue, and we don’t have to work on this.” (2, Field officer)*

These systemic issues hinder timely diagnosis and treatment of leprosy. To mitigate these challenges, experts felt there is a need for increased investment in leprosy control programs. This includes funding for specialized clinics, training of healthcare workers, and procurement of diagnostic tools and medications. Additionally, it was felt that improving the coordination between different levels of healthcare can streamline the referral process, ensuring that patients receive timely and appropriate care.

### Gender and socioeconomic factors

Based on the interviews women were described as having to face additional barriers to access healthcare due to cultural norms, financial constraints, and limited mobility. Socioeconomic factors, such as poverty and lack of education, were further viewed to compound these delays, as individuals may lack the resources or knowledge to seek appropriate care. For example, one of the participants explained:

*“In the more conservative areas, for example, a woman cannot travel alone to see a doctor, she needs a male accompanying her […] the harvesting season, if the family says nobody has now any time to travel, then simply it cannot be done, or the females have to wait until the situation has changed or the activities have become less and somebody has time. […] But, even in the urban areas also, very often females would need the company of a male family member.” (1, Medical director)*

The interviews revealed that women may have limited autonomy to seek medical care, particularly in rural areas where cultural norms restrict their mobility. Financial constraints can also prevent patients from accessing healthcare, as they may prioritize the health needs of their families over their own. These barriers can lead to significant delays in the diagnosis and treatment of leprosy in Pakistan, resulting in more severe disease and disability.

*“People are from very poor areas, they aren’t able to travel from far flung areas to the main referral centres, so they have the financial issues.” (2, Field officer)*

Addressing gender-specific barriers requires targeted interventions that empower women and improve their access to healthcare. This can include community-based programs that provide education and resources for women, as well as initiatives that promote gender equality in healthcare access.

### Health system challenges

The interviews revealed that the health system in Pakistan faces several challenges that contribute to diagnostic delays in leprosy. These include a lack of trained healthcare providers, inadequate diagnostic facilities, and insufficient integration of leprosy services into the general healthcare system. The study highlighted the need for systemic reforms to address these challenges and improve the timeliness and accuracy of leprosy diagnosis. The study also encountered a split in availability of diagnostic capabilities between rural and urban areas, as mentioned by one of the participants:

*“In cities there are a lot of local hospitals and NGOs that are working, but rural side there may be less.” (6, Statistician)*

Inadequate primary diagnostic facilities were also described as posing a significant barrier to timely diagnosis. The study found that many healthcare centres lack the necessary tools and resources to diagnose leprosy accurately, despite there being the capacity for testing nationally. This can lead to delays in diagnosis and treatment, as patients may need to be referred to specialized centres for confirmation. Strengthening the diagnostic capacity of healthcare centres, including the provision of necessary tools and training, is crucial to improving the timeliness of leprosy diagnosis.

*“5 to 6 of such highly qualified laboratories which are basically enough. Okay, not saying there is a lack. […] the point is, as I said, you need a well-functioning referral system.” (1, Medical director)*

The lack of integration of leprosy services into the general healthcare system was also cited as a means of improving diagnosis and treatment. The study found that leprosy services are often siloed, with limited integration into general healthcare programs. This can lead to delays in diagnosis and treatment, as patients may need to navigate multiple systems to receive care. Integrating leprosy services into general healthcare programs was felt to streamline the process and ensure that patients receive timely and appropriate care.

## Discussion

The study highlights a multi-faceted framework of diagnostic delay in Pakistan, involving socio-cultural, professional, and systemic factors. These delays pervade all tiers of the healthcare hierarchy, justifying a multi-level approach to improve general awareness, healthcare education, and organizational structuring. The review highlighted three primary causes of delay: Low population awareness of leprosy; Low levels of professional knowledge about leprosy among healthcare workers; and a lack of program infrastructure.

Recent interventions in India have shown contextualised posters and community meetings as effective interventions to increase the levels of awareness of leprosy within the community [18]. If these interventions could be validated in Pakistan, they could address this element of diagnostic delay. Though evidence is sparse on the levels of knowledge among healthcare workers in Pakistan a 2009 study revealed “inconsistency and deficiencies in the knowledge, referral pattern and cure of leprosy among general practitioners” [19]. To address this aspect of delay the knowledge of healthcare workers must first be quantified and then interventions designed to address the shortcomings. Finally, the experts lamented the lack of national direction in the leprosy programme, future impetus toward delivering a national leprosy programme and targets can address this issue of national infrastructure.

Leprosy experts also highlighted the importance of public health education in changing societal perceptions of leprosy. Misconceptions about leprosy, stigma, and lack of knowledge about symptoms often prevent individuals from seeking timely medical attention. Previous papers in Pakistan have shown strong stigmatizing beliefs about leprosy to exist within certain communities [20]. However, with the low prevalence the research impetus has now decreased and a significant gap in the literature in Pakistan has emerged. As well as being cited by experts as a reason for delay gender-specific barriers to healthcare services in Pakistan are evident in the literature [21]. Further research to understand how gender specifically interacts with leprosy care, and to reveal its impact could help shorten time to diagnosis in the future. Additionally, continuous professional development for healthcare workers is essential to ensure timely and accurate diagnosis of leprosy. Two literature reviews on case detection methods and stigma reduction interventions respectively found zero papers in Pakistan [22,23].

Research to validate these methods in Pakistan could help increase detection rates. Effective management of leprosy requires a well-coordinated healthcare system with adequate resources and specialized facilities [24]. Systemic reforms to address infrastructural deficiencies, including increased funding, specialized facilities, and improved coordination between different levels of healthcare, are also critical to improving leprosy care.

### Recommendations for improving diagnosis and treatment

Our study provides key recommendations for improving leprosy diagnosis and treatment in Pakistan, including raising public awareness, enhancing healthcare provider training, and strengthening healthcare infrastructure. Addressing personal, cultural, gender, and socioeconomic barriers is essential to reducing diagnostic delays.

Public awareness campaigns should target myths and stigma, using culturally appropriate strategies in both rural and urban areas. Engaging local leaders and influencers can promote early diagnosis and treatment. Tailored interventions should focus on high-endemic areas where stigma is more prevalent. Health literacy campaigns must be piloted in diverse communities and monitored through surveys and focus groups to assess their effectiveness.

Healthcare provider training can be improved through ongoing professional development, emphasizing clinical presentation and diagnostic tools. Integrating leprosy education into medical curricula, along with regular refresher courses, will ensure providers stay current. Government collaboration should focus on including leprosy in medical exams, guided by experts, and enhancing existing training programs nationwide.

Strengthening healthcare infrastructure requires sustainable funding for specialized clinics, training, diagnostic tools, and medications. Better coordination within the healthcare system is needed for efficient referrals and timely care. With foreign funding declining, the government must take responsibility for financing and sustaining leprosy services in the long term.

### Limitations and strengths

The study’s qualitative design enabled an in-depth exploration of perceptions and experiences but had limitations, such as a small sample size and potential selection bias, which may affect the generalizability of the findings. Despite this, the study’s strength lies in its detailed thematic analysis and rich data from experienced leprosy stakeholders. Semi-structured interviews allowed for flexibility, enabling deeper exploration of relevant topics based on participants’ responses. The qualitative approach provided depth and context that quantitative methods might miss, offering a comprehensive understanding of factors contributing to diagnostic delays in leprosy.

## Conclusion

The study highlights the need for a comprehensive approach to reduce diagnostic delays in leprosy in Pakistan. Key priorities include enhancing public awareness, improving healthcare professional education, and strengthening the national healthcare infrastructure.

Future research should examine the cultural health beliefs that influence early diagnosis and treatment. Additionally, evaluating the effectiveness of educational campaigns, professional development programs, and systemic reforms will offer valuable insights into improving leprosy care. A coordinated effort involving healthcare providers, policymakers, and community leaders is crucial to addressing the complex factors contributing to diagnostic delays, advancing toward the zero leprosy 2030 target.

## Supporting information

S1 File Interview Guide. (DOCX)

S2 File Consent Form. (DOCX)
