# Supplementary material for: Understanding reasons for delay in diagnosis of leprosy in Pakistan: A qualitative study
Source: PLoS Negl Trop Dis. 2025 Jan 7;19(1):e0012764. doi: 10.1371/journal.pntd.0012764 (PMC11706370; doi:10.1371/journal.pntd.0012764)
Supplement: S1 File — (DOCX) [file pntd.0012764.s001.docx]

**S1 Interview Guide**

[START RECORDING, Zoom]

- Interview [number], date [current date]
- Do you consent that we record this interview, and do you confirm that you have signed a written consent form?

***Semi-structured Questions***

**1. Opening question to introduce participant (icebreaker)**

- Firstly, could you describe your work and background (*urban/rural area, career path etc*.)?
  - Why did you enter the leprosy field?
  - Do you have a personal experience with leprosy, outside of your work?

***Explanation:***

To briefly explain before, we will be splitting the patient trajectory into three segments: before, during and after diagnosis. Firstly, I would like you to focus on the patient’s route to healthcare, before diagnostic measures have been taken.

**2. Access to primary health care facilities in Pakistan (before diagnosis)**

- Please can you describe for me the ideal path a person in Pakistan would take, if experiencing initial leprosy symptoms (anaesthesia/ benign skin changes – but *get them to explain symptoms and grading*)?
  - In which settings should leprosy ideally be diagnosed – (standard primary care facilities, or are outreach programmes/screening also promoted etc.)?
    - *Costs/travel/wrong healthcare services visited e.g. self-med/trad. healing*)? (*unconscious bias – aware of this side?*)
- Is the patient likely to have experienced previous healthcare appointments/multiple referrals, before attending the correct diagnostic facility – (or patients first attempt at diagnosis, where these symptoms were *self-recognised as leprosy*)?
  - In your opinion, how easy is it for patients to recognise leprosy symptoms? (*ease of recognition*)
  - Do you think that the referrals are often appropriate? (*no disease vs late disease*)
- How do you feel about access to healthcare facilities in Pakistan – which factors can affect a person’s ability to seek diagnosis?
  - Structural - *Financial provision/geographical/personnel/symptom awareness*
  - Behavioural - *Age/sex/leprosy type/symptom perception/knowledge/location/education and occupation/stigma/awareness and beliefs/geographic area*
- What recommendations do you have for improving access to leprosy care, pre-diagnosis?

**3. Diagnostic delay (diagnostic ability)**

- How is leprosy typically diagnosed in Pakistan – and is this ideal?
  - Is a clinical examination sufficient, or must biopsy also be performed – how long does this take?
    - *Ease of diagnosis for clinicians – level of training among all specialties?*
- Are there any particular bottlenecks at diagnostic facilities, or their organisation?
  - Personal experiences in diagnosing leprosy or working in diagnostic setting – funding/staffing
- Can you describe the interaction with patients when they enter the clinic/facility?
  - How do patients respond, and what is their demeanour when awaiting diagnosis?
    - *Anxious/relieved to be seen/ignorant/in denial – how might this indicate patient sentiments around healthcare and diagnosis?*
- ‘Previous misdiagnosis’ or ‘high number of consultations’ have been identified as significant sources of delay in diagnosis – do you experience problems in diagnosing patients?
  - Can you explain these types of problems?
  - How can this be fixed?

**4. Preventative measures and treatment (after diagnosis)**

- What happens after a patient has been diagnosed? (*ways in which patient can help to interrupt the diagnostic delay for other patients*)
  - Is ‘contact intervention’ practised in Pakistan? (ex-leprosy patients teaching *community-awareness campaigns*)
  - Is patient treatment started immediately, or is there a significant delay (which may lead to *further disabling*)
- Is active case finding performed via household contact surveys? What do you think about this? (reduce diagnostic delay via reduction in transmission – *break the cycle of transmission/disability burden*)
  - Is post exposure prophylaxis (single dose rifampicin) given to contacts?
- What are the prospects for a newly diagnosed leprosy patient?
  - Career prospects/family and societal treatment/adherence to drug regimen
- Do you think any of the points we just discussed about what happens AFTER diagnosis, such as [give example of what patient mentioned], contribute to creating a delay in diagnosis?
  - If yes, in what way?
    - In your opinion, what is necessary to improve this? (what should be done to decrease the influence of these factors on the delay of diagnosis?)
  - If no, why not?

**5. Closing remarks**

*Recent data on Pakistan shows that 17% of new leprosy cases presented with grade 2 disability – for comparison, India has 250 times the amount of new cases, but only 2% of these are grade 2 disability. – (why is this?)*

- After discussing the patient trajectory through leprosy healthcare, which reasons do you think are most responsible for causing the delay in leprosy diagnosis in Pakistan?
  - Are these more focussed on shaping beliefs before presentation, or on improving diagnostic process, or on improving post-diagnostic actions?
  - Are these more related to systems or individuals/society?
- In your opinion, how can the delay in diagnosis be diminished?
- Do you have any further questions or comments for me?
  - Are there any other topics/issues about the topic of delay in diagnosing leprosy which we haven't discussed yet, and which you would like to discuss?

***[Stop recording]***

Thank you very much for speaking to me today – I’m really grateful for these insights you’ve offered. Would you like to provide any feedback about the interview?

***After interview:***

- Check recording for error
- Collect any notes made
- Transcribe
- Begin analysis with Atlas.ti coding software
