# Supplementary material for: Understanding reasons for delay in diagnosis of leprosy in Pakistan: A qualitative study
Source: PLoS Negl Trop Dis. 2025 Jan 7;19(1):e0012764. doi: 10.1371/journal.pntd.0012764 (PMC11706370; doi:10.1371/journal.pntd.0012764)
Supplement: S2 File — (DOCX) [file pntd.0012764.s002.docx]

**S2 Consent form**

**Certificate of Consent (informed consent)**

**Title**: Reasons for delay in diagnosis of leprosy in Pakistan
**Responsible researcher**: Maxwell Beresford, research intern at MALC and Anil Fastenau, Senior Technical Advisor, Marie Adelaide Leprosy Centre, Pakistan

**To be filled out by the participant:**

I have read through the information provided to me, and I understand the purpose of this study. I have had the opportunity to express any doubts or concerns, and any questions I have asked to the researchers have been answered fully and respectfully. This interview audio will be recorded on an external recording device (with informed consent indicated by a ticked check box), and I am comfortable with my interview responses being analysed anonymously, and with potential publication in the future. I consent to the storing of this research data for a period of 10 years following the last publication, in line with publishing guidelines. I understand that I can withdraw from the study at any time, without providing a reason, and have been assured that my withdrawal will not impact my personal or professional relationship with Marie Adelaide Leprosy Centre. I also understand that I can skip any questions I choose to and may stop the interview at any point I wish.

- I, ....................................... (name of participant), hereby give my consent to be a participant in this study

Date: ...................................................... Signature: ...................................

**To be filled out by the researcher:**

I have ensured, to the best of my ability, that the participant in this interview has been provided with all available information, and I have answered their questions truthfully. The participant has been made aware that the interview will ask for their perceptions of leprosy care in Pakistan, and their responses will be audio recorded, with the data stored for 10 years after the final publication, in line with publishing guidance. I declare that no coercion has been apparent during the gaining of this consent, and that all actions of the participant are voluntary and informed. I confirm that the participant may withdraw their consent at any stage in this study, and shall not be subject to judgement, nor penalty, nor damaged reputation.

Name of researcher: .....................................................................................................

Date: ................................ Signature: ...............................................
